# Carbon‐Based Radar Absorbing Materials toward Stealth Technologies

**DOI:** 10.1002/advs.202303104

**Published:** 2023-09-21

**Authors:** Seong‐Hwang Kim, Seul‐Yi Lee, Yali Zhang, Soo‐Jin Park, Junwei Gu

**Affiliations:** ^1^ Department of Chemistry Inha University 100 Inharo Incheon 22212 South Korea; ^2^ Shaanxi Key Laboratory of Macromolecular Science and Technology School of Chemistry and Chemical Engineering Northwestern Polytechnical University Xi'an Shaanxi 710072 P. R. China

**Keywords:** carbon materials, radar‐absorbing materials (rams), stealth technology

## Abstract

Stealth technology is used to enhance the survival of military equipment in the field of military surveillance, as it utilizes a combination of techniques to render itself undetectable by enemy radar systems. Radar absorbing materials (RAMs) are specialized materials used to reduce the reflection (or absorption) of radar signals to provide stealth capability, which is a core component of passive countermeasures in military applications. The properties of RAMs can be optimized by adjusting their composition, microstructure, and surface geometry. Carbon‐based materials present a promising approach for the fabrication of ultrathin, versatile, and high‐performance RAMs due to their large specific surface area, lightweight, excellent dielectric properties, high electrical conductivity, and stability under harsh conditions. This review begins with a brief history of stealth technology and an introduction to electromagnetic waves, radar systems, and radar absorbing materials. This is followed by a discussion of recent research progress in carbon‐based RAMs, including carbon blacks, carbon fibers, carbon nanotubes, graphite, graphene, and MXene, along with an in‐depth examination of the principles and strategies on electromagnetic attenuation characteristics. Hope this review will offer fresh perspectives on the design and fabrication of carbon‐based RAMs, thereby fostering a deeper fundamental understanding and promoting practical applications.

## Graphical abstract

In past years, outstanding advancements in stealth materials and technologies have built a firm foundation. Carbon‐based radar absorbing materials (RAMs) have certainly become an interesting topic for future development and practical application, with the lead to novel materials. This review covers all the current state of knowledge, the formation and fabrication, and understanding of the microwave absorbing mechanisms, of carbon‐based RAM for radar absorbing properties.

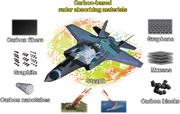

## 1 Introduction

With the advancement in military surveillance systems, stealth technology has attracted significant interest with regard to enhancing survivability in electronic warfare.^[^ ^1^, ^2^, ^3^, ^4^, ^5^, ^6^, ^7^ ^]^ In response to an ever‐increasing range of high‐performance radar‐guided missiles and interceptors on the modern battlefield, the so‐called stealth technology (or low observable technology) has recently begun to be incorporated into the great majority of new aircraft, ship, missile, satellite, and submarine designs to render them invisible to infrared, radar, and other detection methods.^[^ ^8^, ^9^, ^10^, ^11^, ^12^, ^13^, ^14^, ^15^ ^]^ Radar primarily detects aircraft when they enter monitored airspace, i.e., the emitted radar signal is reflected off the aircraft back to the radar. Especially for military aircraft, all new designs take low observable principles and techniques into account, while existing jet fighters are considered for modification in order to reduce their radar signatures.

**Figure 1:**
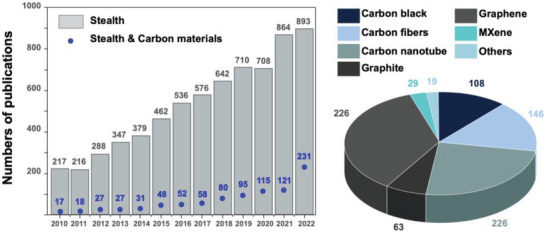
A statistical survey of the number of literature publications per year between 2010 and 2022 based on the keyword “stealth” and other keywords relating to the field of stealth (e.g., “carbon materials”) using the scientific database Web of Science (as of Nov 2022).

The development of stealth technology begun before World War I (1914–1918). At the time, because radar was not yet invented, visibility was the primary concern.^[^ ^16^, ^17^ ^]^ It dates back to 1912 in Germany. Where researchers produced monoplanes in which the fuselage was covered by a transparent material derived from cellulose, and this was painted them with bright colors to further reduce visibility. As a result, the aircraft was invisible from the ground when flown at or above 900 ft and only slightly visible at low altitudes. Soviet aircraft designers in the 1930s also attempted to design transparent aircraft, and radar was invented in 1939. In 1944, a German bomber (Horten Ho 229) was designed, where its wings were made of carbon black‐impregnated plywood held together by a mixture of sawdust and charcoal. Being one of the earliest radar‐stealth aircraft designed by Germans, it absorbed electromagnetic waves and had a small radar cross section. At the same time, Germans used graphite dispersed in a rubber matrix, next to ordinary paper, in a three‐layer material called the chimney‐sweeper and swamp.^[^ ^18^, ^19^, ^20^ ^]^ Application of this rubber‐based composite enabled a reduction in the reflectivity of radio waves. Throughout the 1960s and 1970s, the development of true stealth aircraft continued primarily in the United States (U.S.), with several stealth prototypes being studied. Here, Americans applied similar solutions, but replaced graphite with carbon black (CB) and carbon nanotubes (CNTs) to obtain the Halpern anti‐radiation paint (HARP) used in the aircraft.^[^ ^21^, ^22^ ^]^ With the discovery and development of graphite, CB, CNTs, graphene, graphite nanoplatelets, carbon aerogels, carbon fibers (CFs), and other new macro‐ or nano‐scale carbon allotropes, the carbon family has come to play an irreplaceable role in stealth technology.^[^ ^23^, ^24^, ^25^, ^26^, ^27^, ^28^, ^29^ ^]^ Now, 50 years later, various studies on carbon materials have proved to be important for the future of stealth systems (**Figure** **1**).

**Figure 2:**
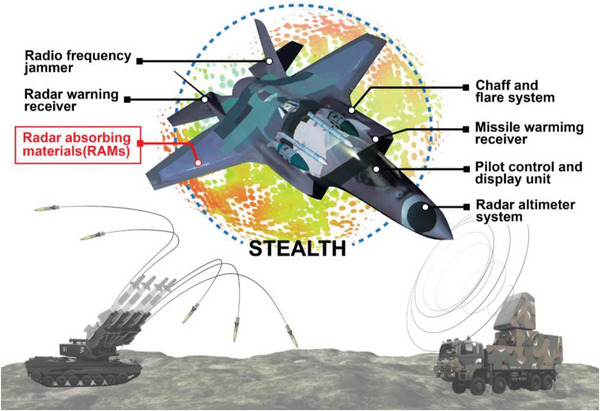
A schematic visualization of the stealth jamming and survival equipment.

Advanced multispectral compatible stealth materials are increasingly needed due to the rapid development of detector and precision guidance technology, which has made the threat to military targets and weapons more serious (**Figure** **2**). Although many research articles are available on the successful realization of Radar Absorbing Structure (RAS) stealth across the world, they mainly focus on the use of Radar Absorbing Materials (RAMs) such as carbon materials as fillers.^[^ ^30^, ^31^, ^32^, ^33^, ^34^, ^35^ ^]^ On the “battlefield” of stealth technology itself, the development of RAMs has been accompanied by continuous advances in the construction of increasingly sophisticated radar as a natural countermeasure.^[^ ^36^, ^37^, ^38^ ^]^

**Figure 3:**
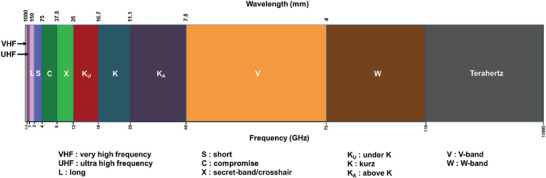
Radar frequency bands according to the Institute of Electrical and Electronics Engineers (IEEE) standards. Reproduced with permission from,^[^ ^43^ ^]^ Copyright Elsevier 2018.

As shown in **Figure** **3**, all radar systems use microwaves (MWs) to detect, locate, and determine the velocity of the target object, and can work across a wide range of transmission frequencies from 3 MHz to 300 GHz and their corresponding wavelengths from 1 mm up to tens of meters depending on the optimum operating conditions.^[^ ^39^, ^40^, ^41^ ^]^ The Institute of Electrical and Electronics Engineers (IEEE) provides additional information on the radar frequency which, in most cases, corresponds to the IEEE standard, as listed in **Table** **1**. The major frequency bands for long‐range surveillance, aerospace, military, and aircraft radars include the L band (1.0–2.0 GHz), the S band (2.0–4.0 GHz), the C band (4.0–8.0 GHz), the X band (8.0–12.5 GHz), the Ku band (12.5–18.0 GHz), the K band (18.0–26.5 GHz), and the KA band (26.5–40.0).^[^ ^42^, ^43^ ^]^ For example, Zhao et al. have explored the possibility of using CNTs as RAMs.^[^ ^44^ ^]^ They observed when the CNT content reaches 10 wt%; the epoxy‐based composites containing CNTs achieved a reflection loss (RL) below –10 dB (90% absorption) over 3.0 GHz in the range of 10.1–13.1 GHz, and the maximum value was –22.89 dB at 11.4 GHz. Additionally, Chen et al. studied the radar‐absorbing effects of graphene in the interlayers of Fe‐doped nanocomposites and found that when the graphene content reached 20 wt% and the film thickness was 2.5 mm, the effective bandwidth was determined to be 14.2 GHz, yielding a maximum RL of −31.5 dB.^[^ ^45^ ^]^ Meanwhile, Wu et al. fabricated polyaniline‐based composites with a 30 wt.% of CBs to obtain a maximum absorption of −40 dB for the frequency range of 9.0–13.0 GHz (the X‐band).^[^ ^46^ ^]^ Similarly, Zou et al. reported a bandwidth below –10.0 dB at 12.2 GHz with a CF content of 0.76 wt.%. Consequently, more and more carbon‐based materials are being used as RAMs in the fabrication of stealth equipment.^[^ ^47^ ^]^

**Table 1:** Radar frequency bands (Reproduced with permission from,^[^ ^43^ ^]^ Copyright Elsevier 2018).

Radar frequency bands (IEEE)	Radar range	Wavelength	Used
TLF	<3 Hz	>100000 km	Frequency of neural activity.
ELF	3–30 Hz	100000–10000 km	Neural activity, communication with submarines.
SLF	30–300 Hz	10000–1000 km	Communication with submarines.
ULF	300–3000 Hz	1000–100 km	Communication with submarines, Communications in mines across the land.
VLF	3–30 kHz	100–10 km	Radio help, underwater communication, wireless pulse gauges, time signals, geophysics.
LF	30–300 kHz	10–1 km	Radio help, AM broadcasting (long wave), RFID, amateur radio, time signals.
MF	300–3000 kHz	1000–100 m	AM broadcasting (medium wave), avalanche beacon, amateur radio.
HF	3–30 MHz	100–10 m	Over the horizon surveillance; low range and low resolution.
VHF	30–300 MHz	10–1 m	Long‐range surveillance, counter stealth, ground‐penetrating; low/medium resolution.
UHF	300–1000 MHz	1–0.3 m	Long‐range surveillance, FOPEN: low/medium resolution.
L	1–2 GHz	30–15 cm	Long‐range air traffic control; medium resolution, small weather effects.
S	2–4 GHz	15–7.5 cm	Terminal air traffic control, long‐range weather observation, moderate weather effect in heavy precipitation.
C	4–8 GHz	7.5–3.75 cm	Long‐range tracking, weather observation, weapon location; increased weather effect in light/medium rain.
X	8–12.5 GHz	3.75–2.4 cm	Short‐range tracking, missile guidance, mapping marine radar, airborne intercept, battlefield surveillance, weapon location.
K_u_	12.5–18 GHz	2.4–1.7 cm	High‐resolution mapping, satellite altimetry, short‐range due to water vapor absorption.
K	18–26.5 GHz	1.7–1.1 cm	Police radar; very limited use due to high water vapor absorption.
K_a_	26.5–40 GHz	1.1–0.75 cm	Short‐range very high‐resolution mapping, airport surveillance; short range due to water vapor absorption.
V	40–75 GHz	0.75–0.4 cm	Scientific remote sensing; high water vapor absorption.
W	75–110 GHz	0.2–0.1 cm	Automobile crusie control (77 GHz), missile seekers, very high‐resolution imaging (94 GHz).

Stealth technologies have improved rapidly over the past 100 years, and will probably continue to develop at a greater speed during the future 100 years if universal properties permit. To the best of the present author's knowledge, the utilization of carbon‐based materials for stealth technologies is still a relatively new topic. Thus, the present review aims to highlight the recent advances in carbon‐based materials as the best feasible option for the development of stealth technologies. To this end, after providing an introduction of electromagnetic waves, frequency bands, radar systems, radar cross section, and radar absorbing materials, a comprehensive discussion of the recent research progress in carbon‐based RAMs, including carbon blacks, carbon fibers, carbon nanotubes, graphite, graphene, and MXene, in terms of electromagnetic attenuation characteristics are provided. Last, this review article addresses the desirable feature of the next generation of carbon‐based RAMs for practical stealth technology.

## 2 Radar Stealth

**Figure 4:**
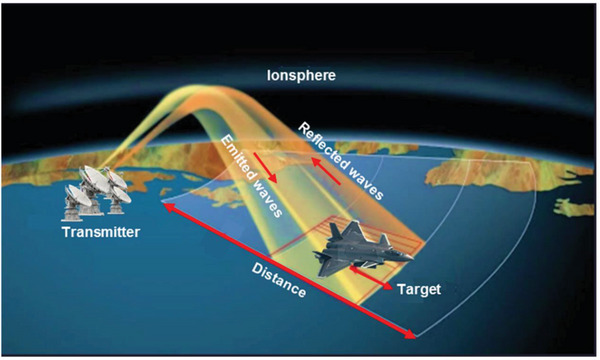
A schematic visualization of the radar operating principle.

Radar absorption is a basic and most important requirement for stealth technologies. Radar stands for the “Radio Detection and Ranging”, first patented by Christian Hulsmeyre in Germany (1904).^[^ ^48^, ^49^, ^50^ ^]^ Radar systems were developed during World War II and their advance continues to the present day. The operating principle of a radar is to send electromagnetic energy pulses in the direction of the target. These pulses move at the speed of light, almost unobstructed, through space until they are encountered by an object.^[^ ^51^, ^52^, ^53^ ^]^ The visualization factors relating to the detection of a target are shown in **Figure** **4**. The main component of the radar system is the transmitter, which can transmit electromagnetic waves in the intended directions. The waves in the transmitter travel through free space, interact with the target under observation, and return to the receiver. Thus, radar measures the interval of time between the generation of the electromagnetic waves and the return of the waves to the radar after reflecting from the target. It uses the radar Equations (1) and (2) to determine the distance between the target and radar as follows.^[^ ^54^, ^55^, ^56^ ^]^:

(1) $$R\;=\frac{ct}{2}\;$$

where *R* is the range between the target and the radar, c is the speed of light, and *t* is the time taken by the energy pulse to move from the radar and back after striking the target, and

(2) $$P_{r}=\frac{P_{t}G_{t}}{4\pi R^{2}}\;\times \frac{\sigma }{4\pi R^{2}}\times A_{e}$$

where *P*_r_ is the returned power, *P*_t_ is the transmitted power, *G*_t_ is the antenna gain, *σ* is the radar cross section (RCS) of the target (m^2^), and *A*_e_ is the effective aperture of the receiving antenna (m^2^). All these variables are used in the detection of an object, but the RCS plays an important role in the detection of the target.

## 3 Radar Cross Section (RCS)

**Figure 5:**
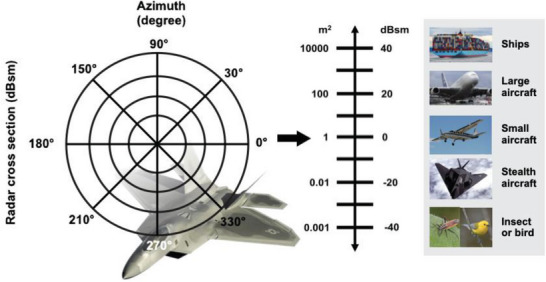
The RCS values of various object or target in square meter and decibel square meter.

The RCS is the virtual size of the object or target as seen by the radar, which is the core detection parameter at radar frequencies. Detailed research on the RCS began in the early 1930s, shortly after the invention of radar.^[^ ^57^, ^58^, ^59^, ^60^ ^]^ The RCS measures the intensity of the waves returning to the radar after reflecting from the object or target. The RCS (*σ*) can be calculated in square meters using Equation (3):^[^ ^61^ ^]^

(3) $$\sigma \;=\lim_{R\to \infty }4\pi R^{2}\;\cdot \frac{lE_{s}l^{2}}{lE_{i}l^{2}}$$

where *E_s_* is the scattered electric field, and *E_t_* is the field incident at the target. Alternatively, the RCS can be calculated in terms of decibel square meters (dB sm) by using Equation (4):

(4) $$\sigma_{\left(dB\;sm\right)}=\;10\log_{10}\;\frac{\sigma_{m^{2}}}{\sigma_{R}}=\;10\log_{10}\frac{\sigma_{m^{2}}}{1m^{2}}\;$$

where σ_R_ is the RCS of a reference object or target with area of 1 m^2^. The typical RCS values of certain objects or targets such as aircraft and ships according to Equations (3) and (4) are indicated in **Figure** **5**. If the RCS is low, then the probability of detection by an enemy during a combat mission is also low, and the capacity for penetration into enemy territory is high. Conversely, a high RCS means that the object or target is likely to be detected by the radar. The factors that influence the RCS of an object or target include:^[^ ^62^ ^]^ i) the size of the target, with a larger target having greater RCS, ii) the material construction and, hence, the reflectivity, of the target, and iii) the geometric shape of the target, which can be designed to prevent the reflection of waves toward the detection system. Among all the factors described above, careful selection of construction materials would be a more viable way for RCS reduction.

## 4 Radar Absorbing Materials (RAMs)

### 4.1 Radar Abosorbing Theory

The RAMs are composites composed of an insulating binder (matrix) along with conductive and magnetic fillers (reinforcements). It acts to reduce the reflected electromagnetic radiations by absorbing a considerable part of the incident electromagnetic energy. It works to reduce the reflected electromagnetic radiation by absorbing a significant portion of the incident electromagnetic energy. This can be achieved by employing one of the following methods. Accepting the signals and subsequently lowering their intensity. This type of action is appropriate for a broad spectrum of radar frequencies. Creating internal reflections of received signals that impede the reflected signals from the outer surface. This action is effective when dealing with a specific frequency.

### 4.2 Radar Abosorbing Mechanism

**Figure 6:**
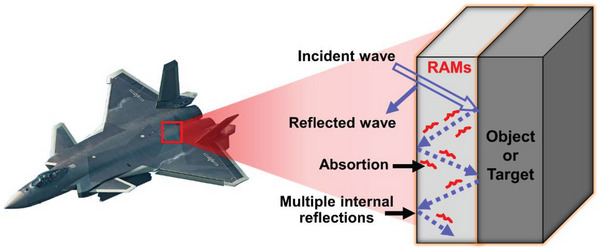
A schematic diagram showing the mechanisms of electromagnetic attenuation by the RAM (i.e., absorption and multiple internal reflections).

The RAMs use two mechanisms to reduce, or eliminate the reflected waves, as detailed below: i) absorption of the electromagnetic wave by the materials, thereby reducing the intensity via dielectric, resistive, and/or magnetic losses, and ultimately converting it to heat energy, and ii) multiple internal reflections of the incident wave from the back and front interior faces of the material.^[^ ^63^, ^64^, ^65^, ^66^, ^67^ ^]^ These mechanisms are shown schematically in **Figure** **6**.

In detail, when electromagnetic energy is incident on a flat lossy material, the reflectivity of the transverse electrically (*TE)* polarized wave (*R_TE_*) is equal to the square of the magnitude of the reflection coefficient (*r*), and is given by Equation (5):^[^ ^68^, ^69^, ^70^ ^]^

(5) $$R_{TE}={\left|r_{TE}\right|}^{2}\;=\;{\left|\frac{\cos\theta \;-\;\mu_{r}^{-1}\sqrt{n^{2}-\;\sin\theta^{2}}}{\cos\theta \;+\;\mu_{r}^{-1}\sqrt{n^{2}-\;\sin\theta^{2}}}\right|}^{2}$$

where *ε*_r_ and *µ*_r_ are the relative complex permittivity and permeability of the lossy material, respectively, and *θ* is the incident angle. Similarly, the reflectivity of the transverse magnetically (TM) and polarized wave (RTM) are also equal to the square of the magnitude *r*, and is given by Equation (6):

(6) $$R_{TM}={\left|r_{TE}\right|}^{2}\;=\;{\left|\frac{\varepsilon_{r}\cos\theta \;-\;\mu_{r}^{-1}\sqrt{n^{2\;}-\;\sin\theta^{2}}}{\varepsilon_{r}\cos\theta \;+\;\mu_{r}^{-1}\sqrt{n^{2}\;-\;\sin\theta^{2}}}\right|}^{2}$$

For normal incidence, the reflectance of the *TE* and *TM* wave decreases as indicated by Equation (7):

(7) $$R\;=\;{\left|\frac{\mu_{r}\;-\;n}{\mu_{r}\;+\;n}\right|}^{2}$$

Additionally, the absorptivity (*A*) may be used, as calculated from Equation (8):

(8) $$A\;=\;1-R\;=\;-{\left|\frac{\mu_{r}\;-\;n}{\mu_{r}\;+\;n}\right|}^{2}$$

Generally, electromagnetic absorption performance can be characterized by the waveguide, coaxial line, and free space methods. The values of ε_r_ and µ_r_ for a given material can then be obtained from the vector network analyzer based on the tested scattering parameters.^[^ ^71^ ^]^ According to transmission line theory, the reflection loss (*RL*) can be calculated using Equation (9):

(9) $$Z_{in}=\;Z_{0}\sqrt{\frac{\mu_{r}}{\varepsilon_{r}}}\;\tanh\left[j\left(\frac{2\pi fd}{c}\sqrt{\varepsilon_{r}\mu_{r}}\right)\right]$$

where *Z*_in_ is the impedance of the absorber, given by Equation (10):

(10) $$RL\;\left(\mathrm{dB}\right)\;=\;20\;\log\left|\frac{Z_{in}\;-\;Z_{0}}{Z_{in}\;+\;Z_{0}}\right|$$

where *f* and *d* are the microwave frequency and thickness, respectively, and *c* is the velocity of light. Meanwhile, *Z*_0_ is the impedance of free space (≈377 Ω), and is given by Equation (11):

(11) $$Z_{0}=\;\sqrt{\frac{\mu_{0}}{\varepsilon_{0}}\approx 377\Omega }$$

where ε_0_ and µ_0_ are the relative complex permittivity and permeability of free space, respectively. Because the impedance (*Z*_in_) of a metallic aircraft is close to zero, a signal that strikes the metallic surface is mostly reflected. Meanwhile, the energy absorbed by the RAM depends on the thickness of the coating, the angle of the incident wave, and the specific material properties. Hence, an RAM must have an impedance value close to that of air and must be able to absorb the energy of the electromagnetic radiation rapidly as it passes through the material. To achieve this goal, the surface of the aircraft must be coated with dielectric or magnetic materials.

## 5 RAMs Based on Carbon Materials and their Composites

### 5.1 Carbon Black (CB)

Carbon black (CB) is an amorphous allotrope of carbon with a low density, high conductivity, and high mechanical strength, and is generally derived from the combustion of petroleum products.^[^ ^72^, ^73^, ^74^, ^75^ ^]^ In particular, it contains more than 90% pure elemental carbon, and is made up of tiny, mostly spherical carbon atoms fused together in clusters known as aggregates. Due to its favorable properties, CB has been the most commonly applied reinforcing carbon filler in the manufacture of composites since the First World War, and is used in applications such as a reinforcing agent to provide mechanical strength to absorber materials. Moreover, the electrical properties of a composite can be enhanced by adding CB with a low particle density (high porosity), small particle size (large surface area), low volatility (few oxygen groups), and favorable structure (better aggregation). This section covers some examples of CB‐based RAMs as composites, single‐ and multi‐layer composites.

**Figure 7:**
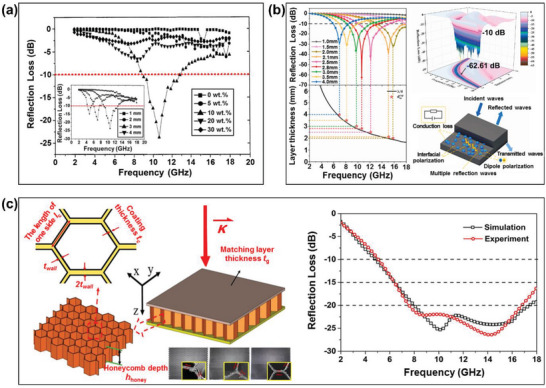
The microwave absorbing properties of various CB‐based RAMs: a) the calculated RL curves for single‐layer silicone rubber composites with CB (Vulcan XC‐72) (Reproduced with permission from,^[^ ^76^ ^]^ Copyright Wiley 2002), b) the calculated RL curve and microwave absorption mechanism of polypropylene‐based composites with various contents of CB (Xyui Zongze) (Reproduced with permission.^[^ ^78^ ^]^ Copyright 2020, Elsevier), and c) the geometry and RL curve of an absorber with a honeycomb sandwich structure (Reproduced with permission.^[^ ^80^ ^]^ Copyright 2019, Elsevier).

Kwon et al. investigated the reinforcing effects of varying compositions (5, 10, 20, and 30 wt.%) of CB (Vulcan XC‐72) fillers in the silicone rubber matrices of single‐layer composites.^[^ ^76^ ^]^ The optimum CB content was identified as 10 wt.%, giving a maximum RL of −22.2 dB in the frequency range of 11.6 GHz at a sample thickness of 1.9 mm (**Figure** **7**a). Similarly, Oh et al. studied the effects of various contents (5, 6, 7, 8, 10, 15, and 20 wt.%) of CB (Vulcan XC‐72) filler in the grass fabric/epoxy prepreg of multi‐layer composites to conclude that the incorporation of 7 wt.% CB significantly enhanced the microwave absorbing properties of the composite, giving an RL of less than 10 dB in the frequency range of 2.4 GHz with a 0.6 mm thick sample.^[^ ^77^ ^]^ Meanwhile, Lee et al. reported the fabrication of polypropylene‐based composites with inner diameters of 3 and 7 mm and various contents of CB (Xyui Zongze) via fused deposition modeling technology, as shown in Figure 7b.^[^ ^78^ ^]^ The optimal CB content was found to be 10 wt.%, giving a maximum RL of –62.6 dB at 10.6 GHz with a 2.8‐mm thick sample. The authors concluded that the microwave attenuation capacity was mainly due to conductive loss, interfacial polarization, dipole polarization, and multiple internal reflections, and that these properties were affected by the distribution of the CB particles. In addition, Mehdizadeh et al. investigated the effects of various CB contents (2, 5, 7, and 10 wt.%) upon the microwave absorbing properties of single‐ and multi‐layer epoxy‐based composites with film thicknesses of 1, 2, and 3 mm in the frequency range of 8.0–12.0 GHz.^[^ ^79^ ^]^ As a result, a maximum RL of –32.1 GHz was obtained in the frequency range of 8.2‐8.8 GHz with a 3‐mm thick multi‐layer sample containing 10 wt.% CB. The authors concluded that a higher content of CB per unit volume enables the attenuation of electromagnetic waves via multi‐scattering and reflection. Meanwhile, Luo et al. used a dipping process to prepare honeycomb cores with distinct pore sizes coated with epoxy resin and filled with conductive CB (Tianjing Ebory), as shown schematically in Figure 7c to obtain a sandwich structure with an RL of up to –2.5 dB within the range of 8.7–16.4 GHz.^[^ ^80^ ^]^

As discussed, an addition of CB alone is instructive to enhancing microwave absorbing properties. Hence, future studies should focus more on the effects of particle size uniformity, film thickness, dispersion, and roughness, while further refining the amount of CB. In addition, methods for skillfully controlling the nano‐scale structure and design of single‐ and multi‐layer materials need to be investigated. Further, the performance of the stealth system could be optimized by enabling the material to absorb radiation without significantly increasing the emissivity.

### 5.2 Carbon Fibers (CFs)

Carbon fibers (CFs) consist of carbon atoms linked in long, thin filaments with diameters in the range of micrometers. Since the 1960s, CFs have been recognized as the most important industrial materials in science and technology.^[^ ^81^ ^]^ They are the preferred material for fiber‐reinforced composites (FRPs) in a wide range of fields, including the automotive industry, mechanical engineering, civil construction, aviation, aerospace, and offshore applications, due to their outstanding properties, such as high specific strength and stiffness, performance‐to‐weight ratio, electrical conductivity, thermal stability, and corrosion resistance.^[^ ^82^, ^83^ ^]^ Most importantly, the use of CFs allows a reduction in the weight of the product or equipment due to its high strength to weight ratio. Due to these properties, CFs are widely used in many applications, including electromagnetic absorber materials.^[^ ^84^, ^85^ ^]^ In this regard, a significant amount of work has been performed on CF‐reinforced polymer composites (CFRPs) and CF/magnetic nanoparticles.

**Figure 8:**
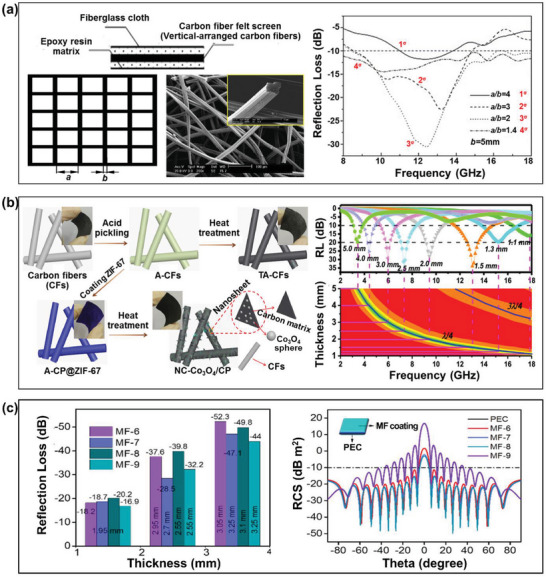
The microwave absorbing properties of CF‐based RAMs: a) a cross section (left) and calculated RL curves (right) of a vertically‐arranged carbon fiber (VACF)/epoxy composite (Reproduced with permission.^[^ ^86^ ^]^ Copyright 2006, Elsevier), b) the schematic fabrication (left) and calculated RL curves (right) of NC‐Co_3_O_4_/CF composites (Reproduced with permission.^[^ ^89^ ^]^ Copyright 2018, ACS), and c) the calculated RL curves (left) and RCS simulated curves (right) of the FeCo/CoFe_2_O_4_/CF composites (Reproduced with permission.^[^ ^90^ ^]^ Copyright 2021, ACS).

For example, Zhao et al. studied the microwave absorbing properties of composites containing inductive activated carbon fiber felt screens (IACFFSs) and vertically‐arranged carbon fibers (VACFs).^[^ ^86^ ^]^ As shown in **Figure** **8**a, a block design method was applied to fabricate the composites with enhanced RAM performances. In this approach, the spacing between the adjacent fibers is important for the microwave absorbing properties of the composite, giving a maximum RL of –30.0 dB in the frequency range of 11.8–18.0 GHz when the interval between strips, the width of the strips, and the fiber space are 5, 8, and 10 mm, respectively. In addition, Ling et al. prepared linear low‐density polyethylene (LLDPE)/ethylene‐octene copolymer composites filled with short CFs via melt blending. The optimum content of short CFs was found to be 30 wt.%, giving maximum RL values of –15.6 dB at 4.6 GHz and –17.4 dB at 16.4 GHz.^[^ ^87^ ^]^ Meanwhile, Ye et al. investigated the effects of coating the CFs with various concentrations of magnetic FeCoNi particles, and reported a maximum RL of –30.6 dB at 11.7 GHz, with an effective bandwidth of 7.4 GHz (from 8.7 to 16.1 GHz) due to the dielectric loss from the magnetic particles.^[^ ^88^ ^]^ In addition, Quan et al. presented the in situ horizontal growth of Co_3_O_4_ nanoparticles embedded in an N‐doped carbon array on the surface of the CFs in carbon paper to fabricate low‐thickness RAMs (NC‐Co_3_O_4_/CP) (Figure 8b).^[^ ^89^ ^]^ Here, maximum RL values of –16.1 and –34.3 dB were achieved at thicknesses of 1.1 and 1.5 mm, respectively. The authors attributed the enhanced microwave absorbing properties of these RAMs to the internal reflections due to dielectric and magnetic losses, the shortened impedance matching gap, the dipolar polarization, and the thickness effect. Moreover, Chen et al. reported the successful fabrication of FeCo/CoFe_2_O_4_/CF hybrid composites via an electrospinning method to obtain a significant microwave absorbing performance, with a maximum RL of –52.3 dB at 5.0 GHz at a sample thickness of only 1.95 mm (Figure 8c).^[^ ^90^ ^]^ In addition, the RCS contribution of as‐prepared composites for EM waves can reach 34.5 dB m^2^. The authors concluded that the EM wave attenuation originates from polarization loss and conductivity loss of the carbon material itself as well as the magnetic loss of the magnetic metal particles.

In brief, a magnetic loss‐based absorbent such as a magnetic metal or metal oxide can be combined with CFs to prepare low‐density composites with strong absorption properties. In this approach, a layer of magnetic powder is deposited on the surfaces of the CFs by chemical doping or surface modification in order to enhance the magnetic permeability of the composites and, thus, obtain excellent microwave absorbing properties. In addition, the CFs can be shaped or arranged to further enhance the electromagnetic absorption.

### 5.3 Carbon Nanotubes (CNTs)

Carbon nanotubes (CNTs) are 1D nano‐scale materials that can be categorized as either amorphous or crystalline on the basis of their structures. Many previous studies have demonstrated that CNTs are 100 times stronger than steel, and one‐sixth as dense.^[^ ^91^, ^92^, ^93^, ^94^ ^]^ In addition, the CNTs have higher thermal conductivities of ≈2000–6000 W m^−1^ K^−1^, while their electrical conductivities are much higher than that of copper.^[^ ^95^, ^96^, ^97^ ^]^ These advantages will become the key to multi‐walled carbon nanotubes (MWCNTs) and single‐walled carbon nanotubes (SWCNTs) in the field of RAMs.^[^ ^98^, ^99^, ^100^, ^101^, ^102^, ^103^ ^]^ This section covers some examples of CNT‐based RAMs as composites, metal‐filled or magnetically coated CNTs, core‐shell CNTs, and CNT/bimetallic metals.

**Figure 9:**
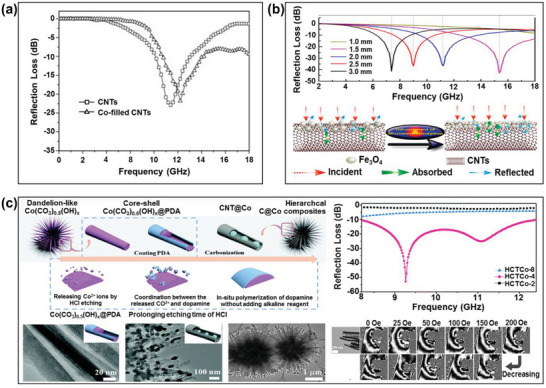
The microwave absorbing properties of CNT‐based RAMs: a) the calculated RL curves of pristine and Co‐filled CNTs (Reproduced with permission.^[^ ^104^ ^]^ Copyright 2010, Elsevier), b) the calculated RL curves (top) and microwave absorbing mechanism (bottom) of magnetic Fe_3_O_4_‐coated CNTs (Reproduced with permission.^[^ ^111^ ^]^ Copyright 2017, ACS), and c) the schematic preparation of core‐shell (Co/CNT) composites (left) and the calculated RL curves (right) (Reproduced with permission.^[^ ^117^ ^]^ Copyright 2020. RSC).

Zhao et al. investigated the effects of Co‐filled CNTs (10 and 20 wt.%) upon the microwave absorbing properties of epoxy‐based composites with thicknesses of 2.22–2.26 mm in the frequency range of 2.0–12.0 GHz.^[^ ^104^ ^]^ The results in **Figure** **9**a revealed that the optimum content of Co‐filled CNTs was 20 wt.%, giving a maximum RL of –21.8 dB at 12.2 GHz. In addition, the effects of various contents (10 and 20 wt.%) of Ag‐filled CNTs upon the epoxy‐based composites were examined, giving a maximum RL of –19.2 dB at 7.8 GHz with 10 wt.% Ag‐filled CNTs and a sample thickness of 1.0 mm.^[^ ^105^ ^]^ In a similar study, Zou et al. studied the effect of different contents (5 and 10 wt.%) of Ni‐filled CNTs in the epoxy‐based composites.^[^ ^106^ ^]^ They concluded that the incorporation of Ni‐filled CNTs at 10 wt.% significantly enhanced the microwave absorbing properties of epoxy‐based composites, which the RL less than –23.1 dB could be obtained in the frequency range of 4.6 GHz from the samples at 2.0 mm of thickness.

The coating of magnetic metals onto the CNT surface is widely used in stealth technology due to the enhancement in the microwave absorbing properties.^[^ ^107^, ^108^, ^109^, ^110^ ^]^ For example, Li et al. prepared Fe_3_O_4_‐coated CNTs to achieve a maximum RL of –43.0 dB and an effective frequency bandwidth of up to 8.5 GHz with a sample thickness of 1.5 mm (Figure 9b).^[^ ^111^ ^]^ In addition, the authors reported that a high surface area in the Fe_3_O_4_‐coated CNTs provides more active sites than the bare CNTs for dissipating and scattering microwaves. Consequently, the composites with the magnetic particle‐coated structures are ideal for achieving a high microwave absorption performance due to the high incidence probability and multiple reflection and absorption of microwaves. Li et al. also deposited CoFe2O_4_ onto the surface of CNTs to achieve a maximum RL of –30.7 dB and an effective frequency bandwidth of up to 12.9 GHz with a sample thickness of 2.0 mm.^[^ ^112^ ^]^ The authors concluded that the enhancement in the absorbing properties of the nanocomposite can be attributed to the improved impedance matching and the synergetic effects of the multiple dielectric and magnetic losses.

Alternatively, due to their core‐shell structures and large surface areas, the filling of CNTs with magnetic metals can prevent the metal nanoparticles from being oxidized and, thus, maintain better stability in practical applications.^[^ ^113^, ^114^, ^115^ ^]^ For example, Qi et al. prepared core/shell structured Fe/CNT nanohybrids via a chemical vapor deposition (CVD) method, to obtain a maximum RL of –40.2 dB at 17.1 GHz with a sample thickness of 1.5 mm.^[^ ^116^ ^]^ Due to the high attenuation constant and good complementarity between the dielectric and magnetic losses, an excellent microwave absorption performance was obtained in the frequency range of 2.0–18.0 GHz. In addition, Wu et al. prepared a high‐performance microwave absorbent by dispersing a considerable amount of Co magnetic nanoparticles within a dandelion‐like CNT assembly, as shown schematically in Figure 9c.^[^ ^117^ ^]^ Due to the decent dispersion and controlled spatial distribution of the Co magnetic nanoparticles inside the CNTs, the composite achieved its full potential for microwave absorption, with an RL of –52.9 dB at 9.3 GHz at a sample thickness of 2.4 mm. In detail, the in situ electric holography and simulation results indicated that the dispersion of the Co nanoparticles at narrow intervals provided a high magnetic loss capacity due to their dynamic coupling network.

**Figure 10:**
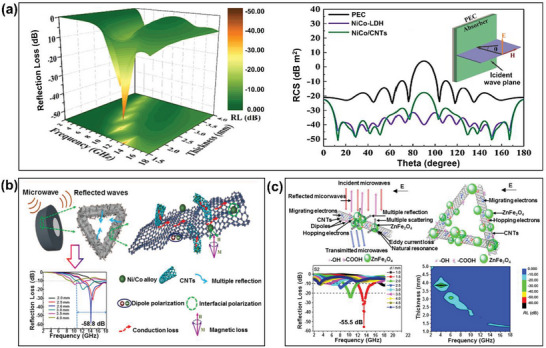
The microwave absorbing properties of CNT/bimetallic metal RAMs: a) the calculated RL curves (left) and RCS simulated curves (right) of the NiCo/CNT composites (Reproduced with permission.^[^ ^121^ ^]^ Copyright 2023, Elsevier), b) a schematic representation of the microwave absorbing mechanism (top) and the calculated RL curves (bottom) of NiCo/carbon nanorod@CNT composites (Reproduced with permission.^[^ ^122^ ^]^ Copyright 2019, ACS), and c) the calculated RL curves (top) and absorbing mechanism (bottom) of ZnFe_2_O_4_/CNT hybrid composites (Reproduced with permission.^[^ ^123^ ^]^ Copyright 2018, Elsevier).

In recent years, CNT/bimetallic metals have gained widespread attention in the design of microwave absorbing materials.^[^ ^110^, ^118^, ^119^, ^120^ ^]^ For example, Xu et al. reported the in situ growing CNTs on the prismatic nickel‐cobalt (NiCo) clusters derived from Ni‐Co layered double hydroxides (NiCo‐LDH) via catalytic carbonization of ethyl acetate.^[^ ^121^ ^]^ The maximum RL of the NiCo/CNT composite was –46.2 dB and the effective absorption bandwidth was 5.8 GHz, with a sample thickness of only 1.5 mm (**Figure** **10**a). In addition, the RCS values of NiCo/CNT composites with metal plate are almost lower than –20 dB m^2^ in the whole range and the RCS reduction can reach 27.5 dB m^2^. Similarly, Wang et al. reported on the fabrication of hierarchical NiCo/carbon nanorod@CNT structures via a carbonization process under an argon (Ar) flow by using Ni‐Co‐MOF‐75 nanorods as precursors.^[^ ^122^ ^]^ Composites with various chemical compositions, densities, and lengths of CNTs were prepared with various Ni^2+^/Co^2+^ ratios of 4:0, 3:1, 2:2, and 1:3, to find that the composite with the 2:2 ratio exhibited an RL of −58.8 dB at 14.0 GHz with a sample thickness of 2.6 mm. The mechanisms behind the outstanding microwave absorbing properties of these NiCo/carbon nanorod@CNT composites are shown schematically in Figure 10b. Thus, the porous structure of the NiCo/carbon nanorod@CNT composite facilitates multiple reflections and scattering of the electromagnetic waves, thereby extending the propagation pathway and enhancing the electromagnetic energy attenuation. In addition, the higher aspect ratio of the carbon nanorods generates conductive losses, while the larger coating density of CNTs provides multiple polarization relaxation processes such as dipole polarization due to the residual oxygen‐containing functional groups (C‐OH and –C(═O)OH), thus further attenuating the electromagnetic energy. Finally, the bimetallic NiCo causes natural and exchange resonances, which also play effective roles in electromagnetic wave dissipation. Meanwhile, Shu et al. reported the successful fabrication of 3D net‐like ZnFe_2_O_4_/CNT hybrid composites via a facile one‐step solvothermal method to obtain a significant microwave absorbing performance, with a maximum RL of –55.5 dB at 13.4 GHz at a sample thickness of only 1.5 mm (Figure 10c).^[^ ^123^ ^]^ The authors demonstrated that the hybridization of magnetic ZnFe_2_O_4_ microspheres with dielectric CNTs influenced both the dielectric and magnetic losses, which determine the impedance matching and electromagnetic wave attenuation. First, the abundant defects and oxygen‐containing functional groups provide a significant quantity of dipoles, which generate polarization relaxation loss in the incident electromagnetic waves. Second, the magnetic microspheres act as bridges for electron‐hopping between neighboring CNTs, as well as generating the magnetic loss via the eddy current loss and natural resonance. Finally, the multiple reflection in the 3D conductive network, and multiple scattering among the interfaces, also play considerable roles in the electromagnetic wave attenuation.

In summary, the CNTs with multifunctional properties have become highly favored among researchers. Consequently, CNTs and CNT‐based composites remain much more attractive than other materials for the fabrication of efficient RAMs. The high specific surface area and high aspect ratio can be very beneficial to the attenuation of microwaves. In addition, the outstanding electrical conductivities of the CNTs in the large‐scale composite can translate into significant conduction‐driven losses for enhanced microwave absorption. These advantages will become the key to the use of CNTs in the field of stealth technology.

### 5.4 Graphite

**Figure 11:**
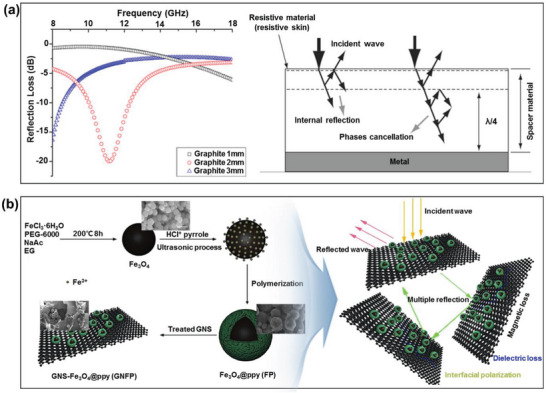
The microwave absorbing properties of graphite‐based RAMs: a) the calculated RL curves (left) and absorption mechanism (right) of SrCoZnFe_16_O_27_/graphite nanocomposites (Reproduced with permission.^[^ ^125^ ^]^ Copyright 2018, Springer), and b) the schematic synthesis (left) and microwave absorption mechanism (right) of graphite nanosheets decorated with core‐shell Fe_3_O_4_@PPy hybrids (Reproduced with permission.^[^ ^130^ ^]^ Copyright 2019, Elsevier).

Graphite is a 3D carbon structure with high conductivity, easy production, low cost, and high stability. These properties have led to numerous investigations on the application of graphite in RAMs. For example, Ismail et al. synthesized single and double‐layer doped CoFe_2_O_4_/graphite composites, and characterized their electromagnetic performances in the frequency range of 8.0–18.0 GHz.^[^ ^124^ ^]^ The CoO and FeO_2_O_3_ precursors were mechanically alloyed and sintered at 800, 900, 1000, and 1100 °C in order to synthesise various particle sizes and shapes. The as‐prepared double‐layer composites revealed significant microwave absorbing performances, with a maximum RL of –15.0 dB at 10.3 GHz at a sample thickness of 2.0 mm. Meanwhile, Rusly et al. studied the microwave absortion properties of single‐ and double‐layer doped SrCoZnFe_16_O_27_/graphite nanocomposites in the frequency range of 8.0–18.0 GHz.^[^ ^125^ ^]^ Here, the SrCoZnFe_16_O_27_ nanoparticles were prepared via solid‐state reaction using high‐energy ball milling, and then sintered at various temperatures (800–1100 °C). As a result, the double‐layer material consisting of SrCoZnFe_16_O_27_ as the absorbing layer and graphite as the matching layer provided a high RL of –19.5 dB at 12.8 GHz with a matching‐layer thickness of 1.0 mm (**Figure** **11**a). The authors attributed this result to the combined effects of material losses and multiple internal reflections at the boundaries.

Graphite materials derived from graphite flakes have also been used in RAMs.^[^ ^126^, ^127^, ^128^, ^129^ ^]^ For example, Su et al. reported the study of a RAM‐based on graphite nanosheets decorated with core‐shell Fe_3_O_4_@PPy hybrids, as shown in Figure 11b.^[^ ^130^ ^]^ The as‐prepared composites exhibited a maximum RL of –49.3 dB at 9.9 GHz at a sample thickness of 2.0 mm. Here, the combined effects of dielectric and magnetic losses could result in enhanced impedance matching, while the introduction of graphite nanosheets generated more interfaces, thereby increasing the interfacial polarization and extending the pathways of the electromagnetic waves via multiple reflections. Similarly, Zhong et al. fabricated a hexagonal boron nitride nanocrystal/graphite nanoflake composite via the wet‐chemistry coating of graphite nanoflakes to achieve a remarkable enhancement in the microwave absorption properties due to the increases in multiple scattering and interfacial polarization, along with the improvement in the electromagnetic impedance matching of the graphite nanoflakes.^[^ ^131^ ^]^ The typical carbonaceous material graphite is considered as one of the most important simple substances, with wide availability in nature. Currently, the graphite is still a hot spot in the field of RAMs where the modification of graphite flake is mainly studied at home and abroad, which nano‐scale graphite and then prepares high‐performance RAMs in combination with other kinds of absorbers.

### 5.5 Graphene

Graphene is a carbon‐based material with a variety of extraordinary properties such as an interconnected porous structure, a large specific surface area, good thermal conductivity, strong chemical resistance, and outstanding electrical conductivity due to its unique band structure.^[^ ^132^, ^133^ ^]^ Graphene‐based materials can solve electromagnetic wave problems with great efficiency, and their microwave absorbing properties give them outstanding potential in the field of RAMs.^[^ ^134^, ^135^, ^136^ ^]^ This section covers some examples of graphene‐based RAMs as composites, monolithic 3D graphene, graphene/dielectric heterostructures, and graphene/dielectric multi‐heterostructures.

**Figure 12:**
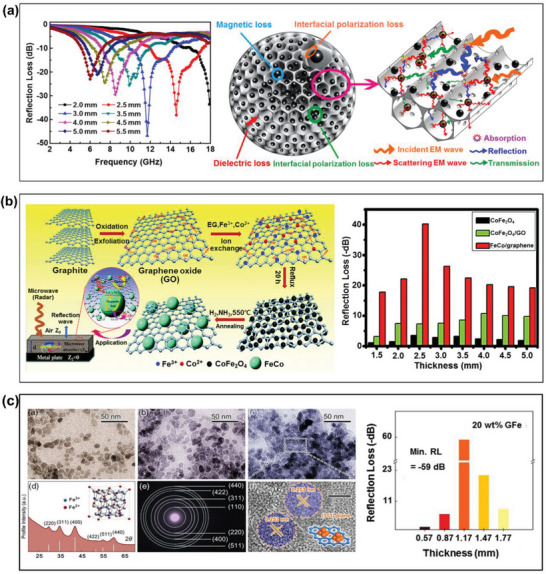
The microwave absorbing properties of graphene‐based RAMs: a) the calculated RL curves (left) and microwave absorbing mechanism (right) of the 3D‐GLN/Fe_3_O_4_ composites (Reproduced with permission.^[^ ^138^ ^]^ Copyright 2021, MDPI), b) the schematic synthesis (left) and calculated RL values (right) of FeCo/graphene composites (Reproduced with permission.^[^ ^140^ ^]^ Copyright 2015, RSC), and c) the microstructure (left) and calculated RL values (right) of N‐doping graphene/Fe_3_O_4_ composites (Reproduced with permission.^[^ ^141^ ^]^ Copyright 2021, Wiley).

Although the unique structure of 2D graphene provides a number of excellent electrical properties, the most challenging issue is that of maintaining these properties at the macroscopic scale. This is mainly hindered by the restacking of the graphene, which leads to destructive aggregation, such that the desired properties cannot be completely realized. Recently, considerable progress has been made toward the fabrication of monolithic 3D interconnected graphenes while maintaining the outstanding properties of single‐layer graphene as well as exhibiting several unique properties. For example, Zhang et al. studied the microwave absorbing properties of monolithic 3D graphene foam (GF) in the frequency range of 2.0–18.0 GHz.^[^ ^137^ ^]^ Here, the GF obtained from graphene oxide (GO) solution was divided into five small batches, four of which were annealed at 200, 400, 600, or 800 °C. Thus, the optimal annealing temperature was found to be 600 °C, giving a maximum RL of –34.0 dB at 13.1 GHz with a sample thickness of 10.0 mm. The dependance of the microwave absorption performance upon the monolithic 3D structure revealed that the GF with a suitable chemical composition and physical structure provides a balance between excellent impedance matching and a high loss characteristic. Meanwhile, Shang et al. reported that the large‐scale fabrication of 3D graphene‐like networks (3D‐GLNs) with hierarchical porous structures by employing an ion‐exchange resin as a carbon precursor.^[^ ^138^ ^]^ The microwave absorbing properties of the Fe_3_O_4_‐loaded 3D‐GLNs were investigated in the frequency range of 2.0–18.0 GHz to reveal a maximum RL of –46.8 dB at 11.8 GHz with a sample thickness of only 3.0 mm (**Figure** **12**a). This excellent microwave absorbing performance can primarily be attributed to the hierarchical structure of the 3D‐GLN/Fe_3_O_4_ composite. First, the 3D network of graphene‐like pore walls provided a certain resistance, which generated induction currents upon electromagnetic incidence, thereby attenuating and transforming the electromagnetic wave energy into thermal energy. Second, the defects and functional groups of the 3D‐GLN not only enhanced the impedance matching, but also introduced electronic dipole polarization relaxation, defect polarization relaxation, and a reduction in the Fermi level, all of which contributed to electromagnetic wave penetration and absorption. Finally, the introduction of Fe_3_O_4_ particles could induce considerable interfacial polarization losses, which originated from the interfaces between the Fe_3_O_4_ and graphene‐like walls.

Typically, the development of graphene/magnetic hybrids is a more direct strategy than the above‐mentioned method because it directly affects the RL values of the composites. For instance, Wang et al. used the atomic layer deposition (ALD) method to fabricate graphene/Fe_3_O_4_ and graphene/Ni hybrid RAMs.^[^ ^139^ ^]^ The results indicated that the graphene/Fe_3_O_4_ composite fabricated by the application of 100 cycles of Fe_2_O_3_ deposition followed by hydrogen reduction provided an optimal RL of –46.4 dB at 15.6 GHz with a sample thickness of only 1.4 mm. This enhancement in absorption performance can be attributed to the effective impedance matching, increased magnetic loss, and multiple interfacial polarization due to the addition of magnetic constituents. The authors concluded that the ALD‐based nano‐scale surface modification of magnetic particles on graphene is a very promising approach to the design of lightweight and high‐efficiency RAMs. Meanwhile, Li et al. fabricated CoFe_2_O_4_/graphene oxide hybrid composites via a facile one‐pot polyol route, and then chemically converted them under H_2_/NH_3_ atmosphere into FeCo/graphene hybrid composites with enhanced attenuation ability for electromagnetic waves in frequency range of 2.0–18.0 GHz (Figure 12b).^[^ ^140^ ^]^ As a result, the maximum RL of the FeCo/graphene composite was −40.2 dB at 8.9 GHz with a matching layer thickness of only 2.5 mm. They concluded that the FeCo/graphene composites exhibited significant microwave absorbing performance enhancement due to remarkable complex permeability from FeCo nanocrystals and permittivity from graphene. In addition, the metallic nature of the FeCo alloy may also contribute to the enhanced permittivity of the FeCo/graphene composites. In addition, Cao et al. reported the N‐doping graphene/Fe_3_O_4_ composites was prepared by a simple hydrothermal method, where GO was reduced into rGO, along with the formation of Fe_3_O_4_ nanoparticles.^[^ ^141^ ^]^ As shown in Figure 12c, the as‐prepared GFe composite provided a maximum RL of –59.0 dB in the frequency range 0–4.14 GHz with a thickness of 1.17 mm. The authors concluded that the benefiting from the synergy between the dielectric genes and the magnetic medium to the enhancement in microwave absorbing properties.

**Figure 13:**
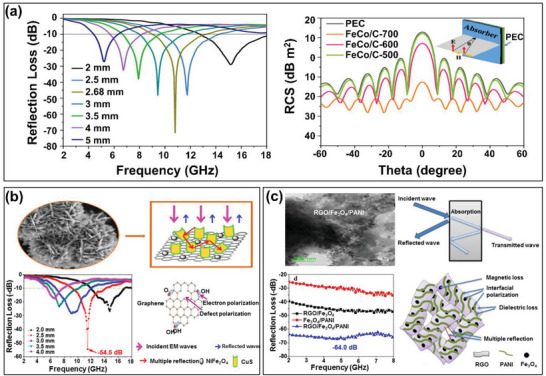
The microwave absorbing properties of graphene/dielectric multi‐heterostructure RAMs: a) the calculated RL curves (left) and RCS simulated curves (right) of the FeCO/graphene composites (Reproduced with permission.^[^ ^142^ ^]^ Copyright 2022, ACS), b) schematic representation of the microwave absorbing mechanism for magnetically decorated graphene/CuS composites (Reproduced with permission.^[^ ^143^ ^]^ Copyright 2016, ACS), and c) calculated RL curves and absorbing mechanism of the graphene/Fe_3_O_4_/PANI composites (Reproduced with permission.^[^ ^144^ ^]^ Copyright 2021, ACS).

To further increase the tunability of the microwave absorbing performance, the construction of graphene/dielectric multi‐heterostructures has been a widely adopted strategy.^[^ ^129^ ^]^ For example, Du et al. studied the microwave absorbing properties of FeCo/graphene composites in the frequency range of 2.0–18.0 GHz.^[^ ^142^ ^]^ The results in **Figure** **13**a revealed that the optimum content of FeCo‐filled graphene was 20 wt.%, giving a maximum RL of –71.63 dB at 10.8 GHz ith a sample thickness of 2.68 mm. In addition, the RCS of FeCO/graphene composite is less than –10 dB m^2^, which provides evidence that the samples have good radar wave attenuation capabilities when the scattering angle is between 0 and 60°. In addition, Liu et al. investigated the effect of CuS and NiFe_2_O_4_‐filled graphene upon the microwave absorbing properties of graphene‐based composites in the frequency range of 2.0–18.0 GHz.^[^ ^143^ ^]^ As shown in Figure 13b, the as‐prepared composites exhibited a maximum RL of –54.5 dB at 11.4 GHz with a layer thickness of 2.5 mm. The authors suggested that the introduction of magnetic particles into the graphene sheets and CuS nanoflakes played an important role in increasing the microwave absorbing properties, not only providing better impedance matching, but also strengthening the ion polarization, dipole polarization, and interfacial polarization. Meanwhile, Manna et al. reported the fabrication of a graphene/iron (II/III) oxide/polyaniline (graphene/Fe_3_O_4_/PANI) composite via a hydrothermal method.^[^ ^144^ ^]^ As shown in Figure 13c, the as‐prepared composite provided a maximum RL of −64.0 dB in the frequency range 2.0–8.0 GHz with a thickness of 0.45 mm. This outstanding response was attributed to the good impedance matching between permittivity and permeability values, as well as magnetic and dielectric losses. Moreover, the high RL of the graphene/Fe_3_O_4_/PANI composite suggested the presence of more interfacial polarization, relaxations, and multiple reflections.

The graphene‐based RAMs have demonstrated their capabilities for high microwave absorbing performance due to the hierachical structure and distinctive electrical properties of graphene and other components. These two properties endow the graphene‐based composites with the greatest potential to achieve high‐performance RAMs. Thus, graphene offers great opportunities for further enhancing the performance of RAMs.

### 5.6 MXene

To further enhance the electromagnetic wave absorption capacity and construct an efficient RAM, researchers have explored the use of new materials such as the family of 2D structures known as the MXenes, which were discovered by Yury Gogotsi in 2011.^[^ ^145^ ^]^ These have the general formula Ti_3_C_2_T*_x_*, where Tx represents an abundant fuctional group such as OH, O, and F, and are considered to be hugely promisng materials for RAMs due to their accordion‐like structures, surface defects, and plentiful surface termination groups. Due to its unique layered structure, hydrophilic surface, high conductivity and good mechanical properties, MXene is widely applied in catalysts,^[^ ^146^, ^147^, ^148^ ^]^ energy storage,^[^ ^149^, ^150^ ^]^ optics,^[^ ^151^, ^152^ ^]^ desalination,^[^ ^153^ ^]^ film,^[^ ^154^ ^]^ and other fields in addition to RAMs. This section covers some examples of MXene‐based RAMs as composites, pure MXene, MXene/dielectric materials, MXene/magnetic materials and MXene/multiple loss materials.

**Figure 14:**
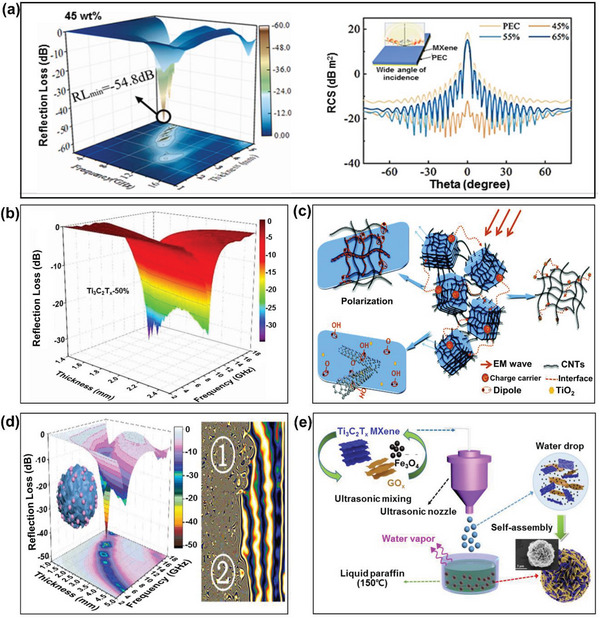
The microwave absorbing properties of MXene‐based RAMs: a) the calculated RL curves (left) and RCS simulated curves (right) of the MXene nanosheets (Reproduced with permission.^[^ ^155^ ^]^ Copyright 2023, Elsevie), b) the calculated R_L_ curves of various MXenes (Reproduced with permission.^[^ ^157^ ^]^ Copyright 2020, IOPscience), c) a schematic illustration of the possible mechanisms of electromagnetic wave absorption in CNT/MXene composites (Reproduced with permission.^[^ ^158^ ^]^ Copyright 2017, RSC), d) the calculated R_L_ curves (left) and computational micromagnetic simulation (right) of MXene/Fe_3_O_4_ composites (Reproduced with permission.^[^ ^160^ ^]^ Copyright 2020, AC), and e) a schematic diagram of preparation of RGO/MXene/Fe_3_O_4_ microspheres (Reproduced with permission.^[^ ^161^ ^]^ Copyright 2021, Elsevier).

For example, Ting et al. prepared MXene nanosheets to achieve a maximum RL of −54.8 dB and an effective frequency bandwidth of up to 3.0 GHz with a sample thickness of 2.0 mm (**Figure** **14**a).^[^ ^155^ ^]^ In addition, the cartesian plot of RCS values that good microwave absorbing performance is exhibited over a wide range of incident angles ranging from –90° to 90°. The authors demonstrated that the MXene nanosheets to tune their EM response properties, a multifunctional MXene which can serve EM wave absorption and shielding performance. Qing et al. prepared Ti_3_C_2_ nanosheets with a typical MXene structure via the treatment of Ti_3_AlC_2_ powders in hydrogen fluoride (HF) and subsequent ultrasonication, and investigated their electromagnetic wave absorptions in the frequency range of 12.4–18.0 GHz.^[^ ^156^ ^]^ The results indicated a maximum RL of –17.0 dB at 14.6 GHz with a matching layer thickness of 1.4 mm, which was mainly attributed to the proper matching of the electromagnetic impedances and suitable dielectric losses. In addition, the high specific surface area and low thicknesses of the Ti_3_C_2_ nanosheets also enhanced the propagation pathway of the incident wave inside the sample. Meanwhile, Zhang et al. investigated the reinforcing effects of Ti_3_C_2_T*_x_* upon the microwave absorbing properties of paraffin‐based composites in the frequency range of 2.0–18.0 GHz.^[^ ^157^ ^]^ The composites were prepared by adding 40, 50, and 60% Ti_3_C_2_T*_x_* into a paraffin matrix with thicknesses of between 1.4 and 2.8 mm. The results indicated a maximum RL of –34.4 dB at 12.0 GHz with a thickness of 1.7 mm (Figure 14b). The authors concluded that the high dielectric loss and multiple reflections between the Ti_3_C_2_T_x_ layers contributed greatly to the enhancement in microwave absorbing properties.

The microwave absorbing performances of the MXenes can be further enhanced via integration with other dielectric loss materials such as carbon‐based materials. For example, Li et al. reported the in situ growth of carbon nanotubes (CNTs) on MXenes via a simple catalytic CVD process.^[^ ^158^ ^]^ The resulting hierarchical CNT/MXene sandwich structure exhibited outstanding microwave absorbing performance (RL max = –52.9 dB at 7.15 GHz) in the 2.0–18.0 GHz frequency range with a low CNT loading of only 35 wt.% and a thickness of only 1.55 mm. As shown in Figure 14c, the CNTs were uniformly distributed on the surface of the MXene flakes, bridging the gap between various MXene particles to form the overall network. The authors reported that this bridging effect provided high conductivity, along with a decrease in the number of surface functional groups, thereby creating more conductive pathways for charge carriers and enhancing the conductivity loss.

Some examples of magnetic loss materials that have been introduced into MXenes include magnetic metals (Fe, Co, Ni), their alloys, and their metal oxides. The high permeability of the magnetic loss material enhances the impedance matching, which is beneficial for the microwave absorbing performance. For instance, Liang et al. reported the in situ growth of uniform and size‐controllable Ni nanoparticles on the surface of MXenes via a novel, facile, and moderate co‐solvothermal method.^[^ ^159^ ^]^ The optimum Ni content was found to be 10 wt.%, giving a maximum RL of –52.6 dB in the frequency range of 8.4 GHz with a sample thickness of 3.0 mm. In addition, Li et al. reported the study of a simple spray‐drying routine to reshape the MXene into a confined and magnetized microsphere with tightly embedded Fe_3_O_4_ nanospheres, contributing to the enhanced specific interfaces and strong dielectric polarization.^[^ ^160^ ^]^ The maximum RL of the MXene/Fe_3_O_4_ composite was –50.6 dB and the effective absorption bandwidth was 4.6 GHz, with a sample thickness of only 2 mm (Figure 14d). The authors concluded that the above result can be attributed to the advantages of the specially designed architecture, especially the synergetic effect between the magnetic units (Fe_3_O_4_) and the dielectric framework (MXene).

The multi‐components in the MXene‐based composites can introduce excellent impedance matching degree and interfacial polarization, and the morphology also has an important influence on microwave absorbing performance. For example, Cui et al. successfully fabricated 3D pleated RGO/MXene/Fe_3_O_4_ microspheres via a simple and rapid ultrasonic spray techonogy to achieve a maximum RL of –51.2 dB at 11.1 GHz with a thickness of 2.9 mm (Figure 14e).^[^ ^161^ ^]^ The synergistic effects between the dielectric elements (RGO, MXene) and magnetic element (Fe_3_O_4_) resulted in good impedance matching and enhanced microwave absorbing properties within the composites. At the same time, the increase in the pore size was conducive to the entry of electromagnetic waves, thereby increasing the probability of electromagnetic wave reflection loss in the material pores.

The MXenes are novel 2D materials with many advantages for use as RAMs, including unique layered structures, high electrical conductivities, and high specific surface areas. The synergistic effects between MXenes and the added dielectric or magnetic materials result in additional loss mechanisms, which can improve microwave absorbing performance. As with graphene, MXenes can be ideal substrates for coupling with other loss materials to further enhance microwave absorbing performance. Thus, the MXenes have wide application prospects in the field of RAMs.

## 6 Summary and the Perspectives

With the increasing interest on stealth materials and technologies, the carbon‐based materials, such as carbon blacks, carbon fibers, carbon nanotubes, graphite, and graphene, are great promising candidates for various radar absorbing materials (RAM) applications as a result of their extremely large specific surface area, low density, excellent dielectric properties, high electrical conductivity, and excellent stability under harsh conditions (**Table** **2**).

**Table 2:** A summary of Carbon‐based RAMs.

Carbon‐based RAMs	Features	Limitations
Carbon blacks	‐ Low cost ‐ Simple substances ‐ Easy to single‐layer and multi‐layer structures ‐ Uniform particles	‐ Difficult to dispersion in matrix ‐ Low microwave absorbing performance ‐ Complex and difficult for optimization process
Carbon fibers	‐ Easy to single‐layer and multi‐layer structures ‐ Moderate microwave absorbing performance	‐ Difficult to dry ‐ High cost ‐ Difficult to use for large‐scale
Carbon nanotubes	‐ High microwave absorbing performance ‐ High electrical conductivity	‐ High cost ‐ Difficult to use for large‐scale ‐ Requires a deep understanding of the interface of multiple structures
Graphite	‐ Low cost ‐ Excellent stability ‐ Easy to use for large‐scale	‐ Possibility of decomposition and mass loss ‐ Low microwave absorbing performance
Graphene	‐ High microwave absorbing performance ‐ High electrical conductivity ‐ Easy to single‐layer and multi‐layer structures ‐ large specific surface area	‐ High cost ‐ Harmful chemicals ‐ Cost for refining ‐ Difficult to use for large‐scale
MXene	‐ High microwave absorbing performance ‐ Easy to single‐layer and multi‐layer structures ‐ Excellent dielectric properties ‐ High electrical conductivity	‐ High cost ‐ Difficult to use for large‐scale ‐ Cost for refining ‐ Complex and difficult for optimization process

**Figure 15:**
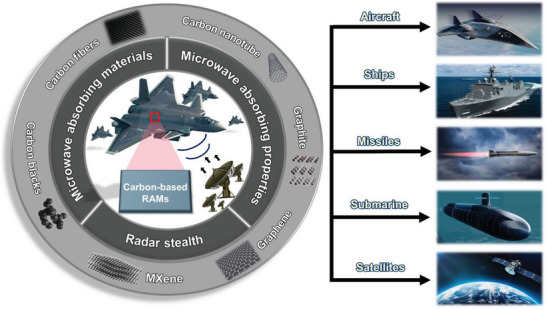
Summary of electromagnetic applications according to carbon‐based materials.

The application of these RAMs could range from the reduction of the radar cross section of the targets, protection and isolation of personnel exposed to electromagnetic waves, electromagnetic isolation for the protection of product or equipment, subjects of interest in the civil and military surveillance fields (**Figure** **15**).

In this review, the recent progress in the uses of these carbon‐based materials for RAMs were comprehensively reviewed. The underlying theories of the radar system, the attenuation of electromagnetic waves, the radar cross section, and radar absorbing material were described. Then, the most recent advances in, and responses of, each type of material and its composites were listed, along with the effects of their fabrication and formation processes, functionalization, and number and thickness of layers upon the electromagnetic attenuation. In addition, the various microwave attenuation mechanisms, such as absorption, dielectric loss, magnetic loss, multiple reflection and scattering, dipole and/or interfacial polarization, and relaxation effects, were discussed and related to the composition and structure of each material, and to its overall attenuation performance. Thus, strategies for using various types of metallic or magnetic particles and/or conductive polymers to enhance the microwave absorption properties via electric and/or magnetic losses were discussed. In particular, the synergies among the various components of the composite materials were shown to enable the tailoring of the permittivity/permeability values, as well as the impedance matching and electromagnetic absorption.

Different strategies for enhancing the microwave absorption properties with magnetic loss using different types of metallic or magnetic particles, as well as conductive polymers have been listed. In addition, the synergies within components that allowed tailoring the permittivity/permeability values, as well as the impedance matching and then the electromagnetic absorption, was emphasized. The role of the composition and structure of the carbon‐based materials that lead to different microwave absorbing mechanisms, such as absorption, dielectric loss, magnetic loss, multiple reflection and scattering microwaves, besides dipole or interfacial polarization and relaxation effects, was related to the microwave absorbing performance of the carbon‐based materials. Based on these recent advances and representative results, the following conclusions and trends for future developments can be noted:

1) Although the excellent electrical conductivities and large surface areas of the carbon‐based materials make them promising candidates for RAMs, the 3D carbon networks are preferred over the 0D, 1D, and 2D carbon networks. This is because the multiple reflections in the 3D carbon network, along with multiple scattering among the numerous interfaces, play considerable roles in enhancing the electromagnetic wave attenuation. Moreover, the optimal microwave absorption performance depends on the physical structure of the 3D carbon network, which can provide a balance between excellent impedance matching and high loss characteristics to deliver excellent microwave absorbing properties.
2) The microwave absorbing performances of multi‐layer structured materials can be limited by disadvantages such as uneven material cross sections or surfaces, non‐uniform particle sizes or dispersions, and incompatibilities between the various layers. Hence, various fabrication strategies such as chemical vapor deposition, magnetron sputtering, or atomic layer deposition have been explored, along with the generation of core‐shell structures, block structures, or foams via wet‐chemistry (e.g., hydrothermal or solvothermal) coating, with some success.
3) At present, the majority of reports have focused on the nano‐ or micro‐structure design of RAMs, which has proven to be effective in considerably enhancing the attenuation of electromagnetic waves. However, the lack of technology for large‐scale fabrication can limit the modularization of these RAMs. Hence, these aspects also need to be considered in order to achieve a better microwave absorbing performance.
4) With the development of national defense and the rise of environmental problems, the use of lightweight materials is becoming increasingly popular. In this respect, the lightweight natures of the carbon‐based materials ensure a balance between stealth and modularization. Nevertheless, while carbon‐based RAMs are being prepared at the present time, their high preparation costs remain an important obstacle that limits their application. This requires expertise from multiple teams and thus needs proper coordination among multi‐disciplinary researchers (material science, nanotechnology, and chemical engineering) and industrialists. The solution to this problem requires expertise from multiple teams and proper coordination between industrialists and multi‐disciplinary researchers in fields such as materials science, nanotechnology, and chemical engineering.

To summarize, the past years have witnessed outstanding advancements in stealth materials and technologies, which have built a firm foundation. Consequently, the carbon‐based RAMs have become a topic of interest for future development and practical application via well‐established or novel materials. Carbon‐based RAMs will play an increasingly important role in many fields in the future. They have great potential for development in areas such as electronic device, artificial intelligence, and military industries. Carbon‐based RAMs, as the newest and fastest growing, will open new avenues for realizing various classes of electromagnetic protection devices.

## Conflict of Interest

The authors declare no conflict of interest.
